# Efficacy and safety of intranasal dexmedetomidine vs. oral chloral hydrate sedation for transthoracic echocardiography in infants with congenital heart disease aged under 3 months: a retrospective study

**DOI:** 10.3389/fped.2026.1840343

**Published:** 2026-06-25

**Authors:** Jingjing Lv, Lihong Jin

**Affiliations:** Department of Anesthesiology, Shanghai Children’s Medical Center, School of Medicine, Shanghai Jiaotong University, Shanghai, China

**Keywords:** chloral hydrate, congenital heart disease, dexmedetomidine, infants, sedation

## Abstract

**Objectives:**

The procedural sedation for young children, especially in infants under 3 months old, and those with congenital heart disease (CHD) is relatively challenging and risky. This study compares the efficacy and safety of intranasal dexmedetomidine and oral chloral hydrate for sedation in these special populations and identifies the risk factors that influencing sedation success.

**Methods:**

This retrospective study included infants with CHD aged under 3 months who underwent outpatient sedation for transthoracic echocardiography (TTE) from January 2023 to January 2024. Sedation was administered as either intranasal dexmedetomidine (Dex) 2 μg/kg or oral chloral hydrate (50 mg/kg). Failure to achieve adequate sedation with the initial dose was defined as initial sedation failure. Ultimate sedation failure was determined if TTE examination remained incomplete despite rescue interventions. Data including heart rate (HR), pulse oxygen saturation (SpO_2_), sedation onset time, discharge time, and adverse reactions were collected. The primary outcomes were the initial sedation success rate. The groups were compared using propensity score matching (PSM) analysis. While the secondary outcome was the risk factors that influencing initial and overall sedation success.

**Results:**

A total of 383 patients were included in the final analysis. The initial sedation success rate and the overall success rate were higher in the Dex 2 μg/kg group than in the chloral hydrate group before and after PSM. The sedation onset time and the discharge time in the Dex 2 μg/kg group was significantly shorter than those in the chloral hydrate group. The decline of HR in the Dex 2 μg/kg group was greater than that in the chloral hydrate group both before and after PSM. No severe adverse reactions occurred. Children with low body weight and prolonged fasting time were independent risk factors that influencing sedation success rate.

**Conclusions:**

Compared with oral chloral hydrate, the use of intranasal dexmedetomidine of 2 μg/kg was related to a significantly higher success rate of sedation without increasing severe adverse events in infants with CHD aged under 3 months. Prolonged fasting time and low body weight may significantly affect sedation success.

## Introduction

In young children and individuals with intellectual disabilities, sedation is often necessary to obtain high-quality biomedical images and to minimize patient stress during echocardiography. For children with congenital heart disease (CHD), particularly those with complex CHD, nervousness or crying can lead to hemodynamic fluctuations and potential instability. Performing echocardiography under such conditions may compromise data acquisition accuracy, which could subsequently influence clinical decision-making.

Various sedative drugs such as pentobarbital, midazolam, chloral hydrate, and ketamine have been used for TTE sedation (1–5). Chloral hydrate, in particular, is a commonly used sedative in many countries. However, in recent years, its application has become increasingly restricted due to potential adverse effects on brain structure and limited availability in some regions (6, 7). Dexmedetomidine, a highly selective agonist for alpha-2 adrenergic receptors, was found to have less impact on respiratory when compared to the majority of other sedative agents and intranasal administration needs minimal cooperation. It is now gaining more and more popularity and is preferred by many physicians because of its noninvasive which is friendly to outpatients without intravenous access. Previous studies in older children have shown that dexmedetomidine has higher success rate and safer than other sedative drugs such as midazolam, pentobarbital, chloral hydrate and ketamine etc (8, 9). Studies also found that dexmedetomidine 2 μg/kg has good sedative effect and appears to be the optimal dose (10). However, research on infants under three months of age, particularly those with concurrent CHD remains limited. This population often presents with complex cardiac malformations, respiratory diseases, other organ dysfunctions, or developmental immaturity, necessitating special attention due to the increased difficulty and risk associated with sedation.

Thus, this study specifically focuses on infants with CHD aged under 3 months. A retrospective analysis was conducted on 383 patients who received either intranasal dexmedetomidine (2 μg/kg) or oral chloral hydrate (50 mg/kg) as the initial sedative, aiming to evaluate the effectiveness of these doses in this population and to identify the risk factors affecting sedation success.

## Methods

### Date collection

This is a retrospective study and was approved by the Ethics Committee of Shanghai Children's Medical Center (SCMCIRB-K2025104-1). The including criteria are as follows: 1) infants (<3 months) who underwent echocardiography between 2023.1 and 2024.1) children who received Dex 2 μg/kg or chloral hydrate 50 mg/kg as initial sedative drugs. Patients with incomplete data and lost to follow-up was excluded from final analysis. Retrospective data including patient age, weight, diagnosis, HR and SpO_2_ before and after sedation, sedation onset and discharge time and adverse reactions were collected. We define simple CHDs as atrial septal defects, ventricular septal defects, patent ductus arteriosus and complex cases as tetralogy of Fallot, pulmonary stenosis, pulmonary atresia, complete atrioventricular canal, double-outlet right ventricle, congenital mitral regurgitation, and others mentioned in our study.

### Sedation procedure

According to the standard sedation procedure of the Joint Committee for International Medical and Health Organization Accreditation (JCI), Patients must fast for a minimum of 2 h prior to the procedure, regardless of breast or formula milk and clear liquids. A pediatric cardiac anesthesiologist routinely evaluates the general data, current medical history, past history, operation history and allergy history of children who present to our institution for TTE before sedation, write a prescription for sedation, and ask the patient's family to sign a sedation informed consent form. Dexmedetomidine or chloral hydrate was selected based on the physician's personal clinical practice, the child's disease status, parental preferences, previous medication history, and the child's level of cooperation. After a comprehensive evaluation of these factors, the sedation plan was determined by the clinicians. With the parents present, a trained pediatric cardiac sedation nurse administered and continuously monitor the children. According to our clinical practice, HR and SpO_2_ were monitored and recorded as standard indicators. Blood pressure (BP) was not routinely used as BP measurements may interfere sedation due to cuff inflation. The state of consciousness, HR, SpO_2_, and were recorded once per 10 min during onset of sedation. Sedation data during TTE examination were captured with automated electronic monitoring. The Ramsay scale was employed to assess the depth of sedation. Successful sedation was defined if the Ramsay sedation scale score was≥5 and the child could receive echocardiography in a sleepy state; Sedation failure was defined as the child failing to fall asleep after drug administration, waking up during the TTE, or the TTE being unable to be completed due to body movement. Then, rescue sedation was needed. Rescue interventions were administered, including 25 mg/kg chloral hydrate, 1 μg/kg dexmedetomidine, or feeding (oral feeding with breast milk or formula as a comfort measure). Onset time was defined as the time from drug administration to the onset of satisfactory sedation (Ramsay score≥5). Discharge time was defined as time from drug administration to regaining consciousness and discharge (modified Aldrete score≥9). In addition, the adverse reaction, such as nausea, vomiting, bradycardia, tachycardia, hypoxia, irritability during regaining consciousness, respiratory depression and respiratory tract obstruction were also recorded. After examination, patients stayed in the post-anesthesia care unit for monitoring. When the modified Aldrete score was ≥9 and no adverse effects were observed, the patient was discharged.

### Statistical analysis

Statistical analysis was performed with SPSS version 23.0 and R version 4.0.4. Categorical variables were expressed as numbers and percentages (%). *χ*2 or Fisher exact tests were used where appropriate. The Shapiro–Wilk test was used to check normality of the continuous data. Continuous variables were presented as the mean ± standard deviation (SD) if normally distributed and compared with Student's *t*-tests. Data that did not follow a normal distribution were reported as medians and interquartile ranges (Q1, Q3), and the Mann–Whitney *U*-test was used for comparisons. *P* values <0.05 were considered statistically significant. PSM analysis was used to account for confounders, including age, sex, weight, fasting time and type of CHD. Multivariable binary regression analysis was performed to determine independent risk factors affecting initial sedation success and overall sedation success of Dex 2 μg/kg.

## Results

A total of 383 patients with diverse diagnosis were included in our final analysis (Group Dex2 μg/kg: *n* = 303; Group Chloral hydrate 50 mg/kg: *n* = 80). After 1:1 PSM, each group comprised 78 patients, and all covariates were well balanced. The primary cardiac diagnosis and demographic characteristics of these children at baseline are shown in Tables 1, 2.

**Table 1:** Primary cardiac diagnosis.

Diagnosis	Count
VSD	131
ASD/PFO	126
TOF, PS	31
AS, COA, SAS, IAA	23
Screening for cardiac murmur	19
TAPVC	16
PDA	11
TGA	7
DORV	7
Vascular Ring	4
PA	2
Cardiac Tumor	2
Cardiomyopathy	2
A-P Window	1
LHLS	1
Total	383

**Table 2:** Patient characteristics.

Variables	Pre-PSM analysis					Post-PSM analysis				
	Total(*n* = 383)	Dex 2 μg/kg(*n* = 303)	Chloral hydrate(*n* = 80)	Statistic	*p* Value	Total(*n* = 156)	Dex 2 μg/kg(*n* = 78)	Chloral hydrate(*n* = 78)	Statistic	*p* Value
Gender				*χ*^2^ = 0.08	0.781				χ^2^ = 0.93	0.336
Female	191 (49.87)	150 (49.50)	41 (51.25)			74 (47.44)	34 (43.59)	40 (51.28)		
Male	192 (50.13)	153 (50.50)	39 (48.75)			82 (52.56)	44 (56.41)	38 (48.72)		
Age(day) M (Q_1_, Q_3_)	42.00 (34.00, 51.50)	43.00 (34.00, 51.50)	40.00 (30.00, 51.25)	*Z* = −1.62	0.106	41.00 (32.00, 53.00)	42.00 (32.00, 53.00)	40.50 (32.25, 51.75)	*Z* = −0.85	0.393
Weight(kg) M (Q_1_, Q_3_)	4.50 (4.00, 5.00)	4.50 (4.00, 5.00)	4.40 (3.90, 5.00)	*Z* = −1.73	0.083	4.50 (4.00, 5.00)	4.50 (4.00, 5.00)	4.45 (4.00, 5.00)	*Z* = −0.96	0.338
Fasting time M (Q_1_, Q_3_)	140.00 (120.00, 172.50)	140.00 (120.00, 177.50)	135.00 (120.00, 162.50)	*Z* = −1.18	0.238	135.00 (120.00, 160.00)	130.00 (120.00, 160.00)	137.50 (120.00, 167.50)	*Z* = −0.64	0.525
Type of CHD				χ^2^ = 1.18	0.278				χ^2^ = 0.12	0.725
Simple cases	277 (72.32)	223 (73.60)	54 (67.50)			110 (70.51)	56 (71.79)	54 (69.23)		
Complex cases	106 (27.68)	80 (26.40)	26 (32.50)			46 (29.49)	22 (28.21)	24 (30.77)		

Z: Mann–Whitney test, χ²: Chi-square test.

M, Median; Q_1_, 1st Quartile; Q_3_, 3st Quartile.

There was no significant difference of gender, age, weight, fasting time and type of CHD between Dex 2 μg/kg and chloral hydrate 50 mg/kg group before and after PSM (*P* > 0.05) indicating that the populations in both groups were comparable.

In the Dex 2 μg/kg group, the total number of successful sedations was 283 cases, of which 21 were rescue successes (*n* = 11 successful rescue by feeding, *n* = 10 successful rescue by an additional dose of 25 mg/kg chloral hydrate). In the chloral hydrate group, the total number of successful sedations was 54 cases, of which 5 were rescue successes (*n* = 4 successful rescue by feeding, *n* = 1 successful rescue by adding 1 μg/kg dexmedetomidine). The overall success rate for Dex 2 μg/kg before and after PSM was 93.4% and 97.4%. The initial sedation success rate for Dex 2 μg/kg before and after PSM was 86.5% and 85.9% respectively. Both the overall success rate and the initial success rate of Dex 2 μg/kg were higher than the chloral hydrate group (*P* < 0.01). The sedation onset time for Dex 2 μg/kg was 15(15,20) and 15(7,22.75) minutes before and after PSM. The discharge time for Dex 2 μg/kg was 90(60,115) and 87.5(55,110) minutes before and after PSM (Table 3). Both the onset time and discharge time of Dex 2 μg/kg were shorter than the chloral hydrate group (*P* < 0.01). The decline of HR for Dex 2 μg/kg was 18(15,24) and 30(14,37) before and after PSM, which was significantly higher than chloral hydrate group (*P* < 0.05). There was no significant difference of SpO_2_ changes between the 2 groups (*P* > 0.05). There was one case where HR change decrease exceeded 30%, but it resolved spontaneously without requiring atropine. 9 cases of SpO_2_ changes exceeded 5% with all SpO_2_ values were greater than 93 in group Dex 2 μg/kg (Table 4). Symptom resolution was achieved after physical stimulation or neck extension beyond neutral, with no other clinical intervention such as face mask oxygen delivery, insertion of an oral or intranasal airway or invasive manipulation required. No severe adverse reactions were observed in this study.

**Table 3:** Onset and discharge time.

Variables	Pre-PSM analysis				Post-PSM analysis			
	Total(*n* = 383)	Dex 2 μg/kg(*n* = 303)	Chloral hydrate(*n* = 80)	*p* Value	Total(*n* = 156)	Dex 2 μg/kg(*n* = 78)	Chloral hydrate(*n* = 78)	*p* Value
Initial success rate		262 (86.5)	49 (61.3)	<0.001		67 (85.9)	49 (62.8)	0.002
Overall success rate		283 (93.4）	54 (67.5)	<0.001		76 (97.4)	54 (69.2)	<0.001
Onset time								
M (Q_1_, Q_3_)		15 (15,20)	25 (15,30)	<0.001		15 (7,22.75)	25 (15,30)	<0.001
Discharge time								
M (Q_1_, Q_3_)		90 (60,115)	100 (70,120)	0.007		87.5 (55,110)	100 (70,120)	0.013

M, Median; Q_1_, 1st Quartile; Q_3_, 3st Quartile.

**Table 4:** Vital signs during sedation.

Variables	Pre-PSM analysis				Post-PSM analysis			
	Total(*n* = 383)	Dex 2 μg/kg(*n* = 303)	Chloral hydrate(*n* = 80)	*p* Value	Total(*n* = 156)	Dex 2 μg/kg(*n* = 78)	Chloral hydrate(*n* = 78)	*p* Value
Change of HR								
M (Q_1_, Q_3_)		18 (15,24)	12 (5,19)	0.001		30 (14,37)	19 (9,31)	0.034
Change of SpO2								
M (Q_1_, Q_3_)		1 (0,3)	1 (0,3)	0.072		1 (0,3)	1 (0,3)	0.093

M, Median; Q_1_, 1st Quartile; Q_3_, 3st Quartile.

As shown in Table 5, we analyzed the associated risk factors that influencing initial and overall sedation success of Dex 2 μg/kg. We found that prolonged fasting time and weight were independent risk factors influencing both initial and overall sedation success. (Weight, OR 1.753, *P* < 0.05 and OR 2.117, *P* < 0.05; Fasting time, OR 0.992, *P* < 0.05 and OR 0.989, *P* < 0.05). While gender and age and type of CHD were not independent risk factors influencing sedation success rate.

**Table 5:** Risk factors affecting initial sedation success and overall sedation failure of Dex 2 μg/kg.

Variables	Initial sedation success			Overall sedation success		
	OR	Cl	*p* Value	OR	Cl	*p* Value
Gender	1.135	0.567–2.272	0.720	1.199	0.452–3.183	0.715
Age	1.002	0.981–1.024	0.842	1.001	0.972–1.031	0.935
Weight	1.753	1.049–2.929	0.032	2.117	1.026–4.371	0.042
Fasting time	0.992	0.985–0.999	0.018	0.989	0.980–0.997	0.011
Type of CHD	1.175	0.510–2.709	0.705	1.436	0.434–4.754	0.554

## Discussion

In this retrospective cohort study, we demonstrated that intranasal dexmedetomidine was associated with a higher sedation success rate compared with oral chloral hydrate for infants with CHD aged < 3 months, without observed severe adverse events. Prolonged fasting time and low body weight were independent risk factors that influencing initial and overall sedation success.

The primary goal of outpatient sedation is to achieve a high success rate, which can alleviate pediatric distress and improve parental satisfaction. However, the success rate of procedural sedation is highly associated with the depth of sedation to gain control of the behavior of uncooperative children, and the increased risk of desaturation during deep sedation would interrupt examinations and decrease success rates in the end (9, 11). For infants ≤3 months who presents unique challenges due to immature hepatic/renal function, heightened sensitivity to respiratory depression, and narrow therapeutic windows, especially for those children with complex CHD and cardiopulmonary maldevelopment. Pharmacological choices and dosing require extreme caution. It is more difficult and important to select safe and effective sedative drugs. In previous studies, traditional agents like midazolam (12, 13), phenobarbital, chloral hydrate (14), ketamine (5), and propofol all have been reported to be used in this age group but they are associated with significant limitations, including unpredictable efficacy, profound respiratory or cardiovascular depression, and potential toxicity. Dexmedetomidine, a highly specific and effective *α*_2_-adrenergic receptor agonist, can directly bind to the *α*₂-adrenergic receptor in the locus coeruleus to activate the endogenous sleep pathway. The sedative dose of dexmedetomidine can establish a natural sleep state, which indicates that the sedative dose of dexmedetomidine has little effect on respiratory depression. Moreover, previous studies have demonstrated that dexmedetomidine displays neuroprotective effects in animals and humans and can inhibit neuronal apoptosis (15, 16). Furthermore, intranasal use of dexmedetomidine can avoid first-pass hepatic metabolism and can be rapidly and efficiently absorbed with better acceptability in children. These advantages are particularly important to young infants with immature hepatic function and ongoing brain development. In our present study, we retrospectively demonstrated that intranasal dexmedetomidine (2 μg/kg) is effective for sedation in infants with CHD under 3 months of age without severe adverse events observed. Previous studies have demonstrated that children sedated with dexmedetomidine displayed statistically higher successful sedation rate and lower incidence of respiratory depression or oxygen desaturation than those sedated with the other sedatives (11, 17). In our study, the initial sedation success rate of dexmedetomidine (2 μg/kg) was 86.5% which is similar to previous study. Due to the low initial sedation success rate in the chloral hydrate group, rescue sedation regimens were required, which resulted in a longer overall onset time and a prolonged discharge time. Therefore, dexmedetomidine had a relatively faster onset of action, enabling pediatric patients to be discharged home earlier. These results support that dexmedetomidine may be a better choice for sedation during echocardiography in infants under 3 months of age.

Due to its numerous advantages, such as no significant respiratory depression which holds particular value for infants <3 months, and its ability to provide cooperative sedation, dexmedetomidine has been increasingly favored in pediatric sedation in recent years. However, compared to traditional sedatives, it has a higher incidence of inducing bradycardia, which has raised clinical concerns. The mechanism involves that its activation of *α*₂ A-adrenoceptor subtypes in the central nervous system (specifically the locus coeruleus), which inhibits the release of norepinephrine, thereby reducing central sympathetic tone and causing decreased HR. Importantly, in infants < 3 months, the blood-brain barrier and autonomic regulatory functions remain underdeveloped. This immaturity allows the drug to more readily cross the blood-brain barrier, potentiating its effects and leading to bradycardia. The HR decrease caused by dexmedetomidine is usually 10% to 30% of the baseline value which may be influenced by different disease and physical status. There are also studies revealed that patients with dexmedetomidine continuously infused present high incidence of bradycardia while a single dose of dexmedetomidine induces fewer changes in HR and compared to intravenous administration, intranasal administration is associated with a less pronounced decrease in HR, making it a more favorable delivery route. In our study, dexmedetomidine was administrated intra-nasally, the HR decreases were more significant than sedation with oral chloral hydrate. The median decrease rate was higher than that reported in most studies. This finding may be attributed to the younger age of our study population, reflecting increased sensitivity to dexmedetomidine and a heightened propensity for bradycardia in infants under 3 months of age. Therefore, clinicians should be aware that dexmedetomidine administration in infants younger than 3 months warrants particular caution, especially in those with atrioventricular block or severe valvular regurgitation.

In our study, we found that prolonged fasting time were independent risk factors that influencing sedation success rate. This is consistent with a prior report from our center involving CHD patients with Down syndrome (17). It is speculated that the reasons are attributed to that extended fasting increases gastric pH and reduces motility, impairing absorption of intranasal dexmedetomidine and fasting-induced physiological stress (hunger, anxiety, dehydration) elevates endogenous catecholamines, counteracting dexmedetomidine's central sympatholytic effects. Further studies should be conducted to validate this in future. Prolonged fasting not only reduces sedation success rate, but also puts patients in a dangerous state because prolonged fasting leads to decreased blood volume and increased hemodynamics fluctuations in younger patients with complex congenital heart defects. Therefore, optimization of fasting protocols, individualized dosing strategies, and enhanced monitoring are essential in this population.

## Conclusion

In conclusion, intranasal dexmedetomidine compared with oral chloral hydrate provides relatively more efficient and effective sedation without observed severe adverse events for echocardiographic imaging in infants with CHD under 3 months of age. Prolonged fasting time and low body weight were independent risk factors that influencing initial and overall sedation success.

## Data Availability

The original contributions presented in the study are included in the article/Supplementary Material, further inquiries can be directed to the corresponding author/s.
